# A Successful Pregnancy Following Intracytoplasmic Sperm Injection in a Breast Cancer Survivor: A Case Report

**DOI:** 10.7759/cureus.55756

**Published:** 2024-03-07

**Authors:** Nancy Nair, Akash More, Brij Raj Singh, Achyut Wadkar, Priyal Tilak

**Affiliations:** 1 Clinical Embryology, School of Allied Health Sciences, Datta Meghe Institute of Higher Education and Research, Wardha, IND; 2 Clinical Embryology, Datta Meghe Institute of Higher Education and Research, Wardha, IND; 3 Anatomy, Datta Meghe Institute of Medical Science, Wardha, IND; 4 Clinical Embryology, School of Allied Health Sciences, Datta Meghe Institute of higher Education and Research, Wardha, IND

**Keywords:** intra-cytoplasmic sperm injection, sperm dna fragmentation, leucocytospermia, ductal carcinoma, primary infertility

## Abstract

This report documents the case of a 36-year-old female diagnosed with stage I invasive ductal carcinoma of the left breast who, alongside her 39-year-old husband, sought fertility assistance at our center due to primary infertility. Having survived cancer twice in the span of their seven-year marriage, the couple faced the challenge of overcoming both the repercussions of cancer treatment and difficulties in conceiving. Initial attempts through three intrauterine insemination (IUI) cycles proved unsuccessful, leading the couple to opt for in vitro fertilization (IVF). The fertility assessment of the husband revealed the presence of several pus cells and a high sperm DNA fragmentation index (DFI). To address this, a medication regimen was administered to improve sperm quality. Concurrently, the female underwent controlled ovarian stimulation (COS) with the anti-estrogen agent letrozole to mitigate the risk of estrogen surges that could compromise her health. Subsequently, oocytes were retrieved from the female, and intracytoplasmic sperm injection (ICSI) was used to facilitate fertilization with her husband's sperm. Following successful embryo development, the patient underwent embryo transfer (ET), resulting in a positive beta-human chorionic gonadotropin (beta-hCG) result, signifying a successful conception.

This case report highlights the intricate challenges faced by individuals with a history of breast cancer, emphasizing the delicate balance required in managing infertility in such circumstances. The described approach, involving personalized treatments and meticulous care, underscores the possibility of achieving successful conception for females struggling with fertility issues post-cancer survival. The documented journey serves as a testament to the resilience of individuals facing the dual challenges of cancer survival and infertility, offering insights into the complexities of their reproductive healthcare.

## Introduction

With 2.3 million new cases detected annually, breast cancer is one of the most common cancers in women [1]. It is the second most frequent non-skin cancer overall, accounting for 10.4% of all cancer incidences in women (behind lung cancer). It also ranks sixth in terms of cancer mortality among women [2]. While men are affected by breast cancer approximately 100 times less frequently than women, they typically experience more adverse outcomes due to late detection. Cancer cells share many characteristics with the cells of the host organism, including similar (though distinct) DNA and RNA. This similarity makes them less likely to be detected by the immune system, particularly if it is weakened [3]. An enlargement or lump in the breast that does not feel like the surrounding tissue may be one of the symptoms or indicators of breast cancer [4], as well as changes in breast size, shape, and appearance that include the skin, such as dimpling on a breast. Breast lumps can occur due to multiple reasons, most of which are non-malignant [5]. Breast anomalies that are not cancerous include benign tumors such as cysts and fibroadenomas, as well as infections. Breast cancer might also spread to other parts of the body and cause new symptoms. The lymph nodes under the arm are usually the first to show signs of spread, but they are also home to inaccessible malignant lymph nodes [6].

There are various forms of breast cancer, with ductal carcinoma being the most prevalent type. A kind of breast cancer known as invasive ductal carcinoma (IDC), also known as infiltrating ductal carcinoma, begins in the milk ducts of the breast and spreads to nearby tissue. IDC can "metastasize," or spread, by passing through blood vessels or lymph nodes to other areas of the body [7]. It has been determined that this constitutes metastatic breast cancer. It is the most prevalent kind of breast cancer, accounting for nearly 80% of cases. It is also the most prevalent kind of breast cancer in men. Following a diagnosis, IDC is often discovered with standard breast cancer testing, like mammography [8]. Even if they do not necessarily cause the classic signs of breast cancer, a new lump or other unexpected changes in the breast can be a predictor of IDC. If the physician suspects breast cancer, further testing and a breast biopsy may be required to confirm the diagnosis of IDC.

The staging of breast cancer is based on the size of the primary tumor and the degree of disease metastasis. The location, size, and degree of unchecked proliferation also determine the stage of cancer, which indicates the disease's progression [9]. Ductal carcinoma progresses through five distinct stages. In stage 0, the malignancy is localized within the milk ducts, often referred to as "non-invasive ductal cancer in situ. As it advances to stage 1, the cancer extends beyond the ducts into the surrounding breast tissue but has not yet reached the lymph nodes. Stage 2 is characterized by the presence of a small tumor, which may involve one to three lymph nodes or a larger tumor that has not migrated to any lymph nodes. Moving to stage 3, the cancer has not yet spread to other body parts but has frequently extended to more than three lymph nodes or is causing infection and redness over a substantial portion of the breast surface. Finally, in stage 4, the cancer has metastasized to distant organs, such as the bones, liver, lungs, brain, chest wall, or distant lymph nodes [10].

In cases of hereditary breast and ovarian cancer, BRCA1 and BRCA2 genes often exhibit genetic variations. Approximately 3% of breast cancers (affecting 7,500 women annually) and 10% of ovarian cancers (impacting 2,000 women annually) are attributed to hereditary variations in these genes [11]. BRCA1 and BRCA2 genes play a crucial role in suppressing the development of specific malignancies. An individual is predisposed to inheriting these genetic abnormalities, leading to an increased risk of developing breast, ovarian, and other cancers, as the abnormal DNA sequences in BRCA1 and BRCA2 impede their normal functioning [12]. Reproductive-aged breast cancer patients commonly present with biologically aggressive disease at diagnosis, often necessitating adjuvant cytotoxic therapy. Such treatments may jeopardize gonadal function and pose a threat to future fertility. While the incidence of chemotherapy-induced ovarian failure can be estimated based on factors like the patient's age, the type of regimen administered, and cumulative dosage, accurately predicting the risk of future sterility remains challenging. Among the drugs frequently employed in breast cancer treatment, alkylating compounds pose the highest risk of gonadotoxicity. Taxanes induce moderate ovarian damage, with lower toxicity risks associated with methotrexate and 5-fluorouracil. In such cases, a thorough evaluation and personalized interventions are imperative to optimize a couple's chances of achieving a successful pregnancy [13].

## Case presentation

**Figure 1:**
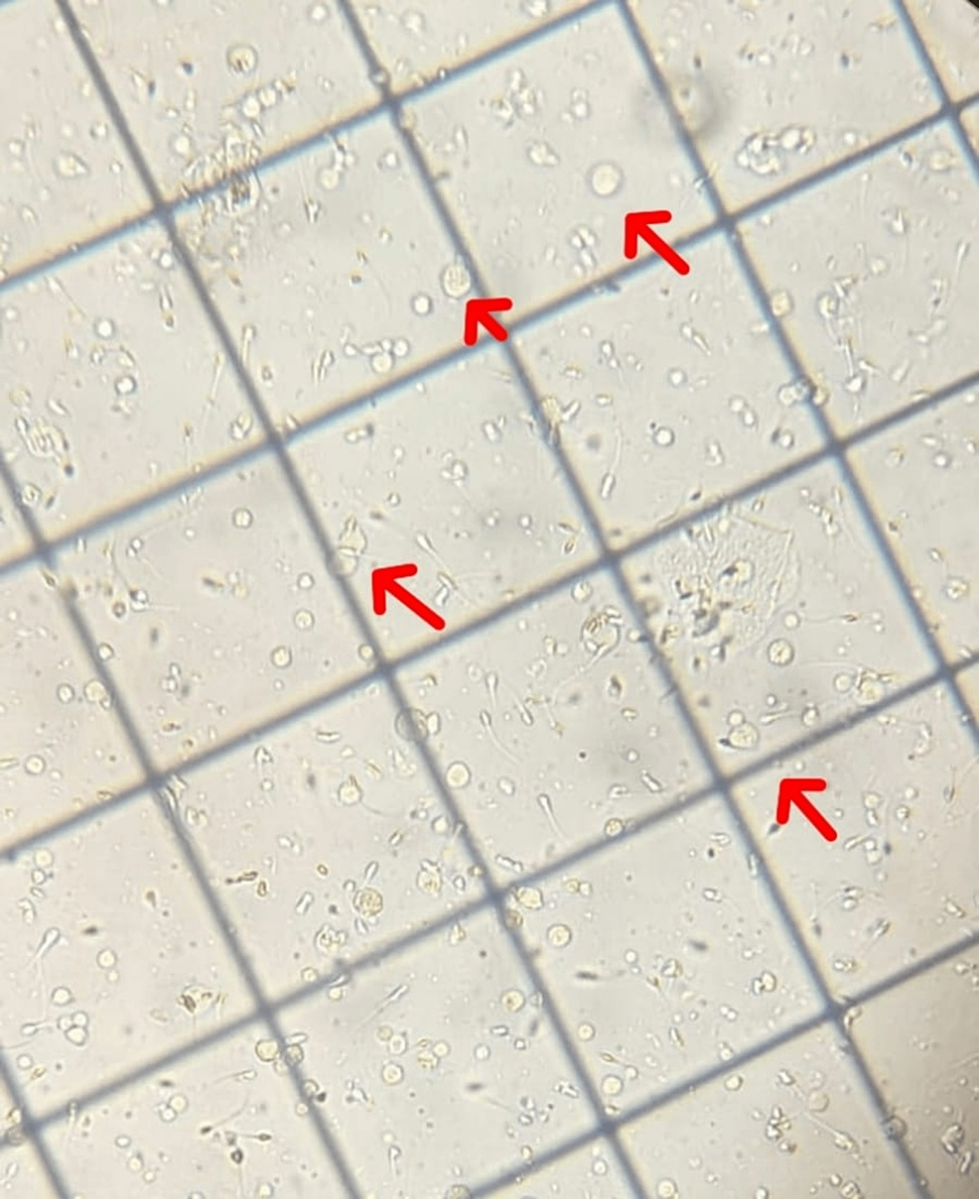
Presence of leucocytes in the semen sample The red arrow shows the leucocytes

The patient was a 36-year-old female experiencing infertility who was referred to our fertility clinic along with her 39-year-old husband. The couple had been married for seven years, facing primary infertility as they had been unsuccessful in conceiving. The female was a survivor of stage I invasive ductal carcinoma of the breast, initially diagnosed in 2015. Following the cancer diagnosis, she had undergone multiple rounds of treatment, including chemotherapy, endocrine therapy, and a biopsy. Post-recovery, the couple had tried conceiving naturally for more than a year but failed. After detailed counseling regarding IVF, the couple opted for three in vitro fertilization/intracytoplasmic sperm injection (IVF/ICSI) cycles. Before IVF, they also underwent three cycles of intrauterine insemination (IUI), all of which were unsuccessful. An assessment of the husband's semen analysis revealed an elevated number of pus cells, indicative of leucocytospermia (Figure 1), and a high sperm DNA fragmentation index. Apart from occasional smoking, the husband had no history of drug exposure, excessive alcohol consumption, or ongoing medical concerns. He reported no testicular injury, varicocele, STDs, or a history of cryptorchidism. Notably, the couple did not have a familial history of infertility or related issues.

History

The patient presented with invasive ductal carcinoma, a moderately differentiated stage I tumor diagnosed at the age of 32 years. Hormone receptor analysis revealed negative estrogen receptor (ER) status (96%) and positive progesterone receptor (PR) status (29%). Additionally, genetic analysis confirmed the presence of a BRCA mutation. Neoadjuvant chemotherapy was initiated, and a small breast tissue sample was subjected to laboratory testing. The report indicated ER/PR-positive status (IHC ER: 100%, PR: 1%) and HER2-positive expression (IHC HER2: 0, HER2 FISH ratio at 2.3). Consequently, adjuvant chemotherapy included trastuzumab and pertuzumab over six cycles. Pathologically, the patient was staged as T1N0M0.

**Figure 2:**
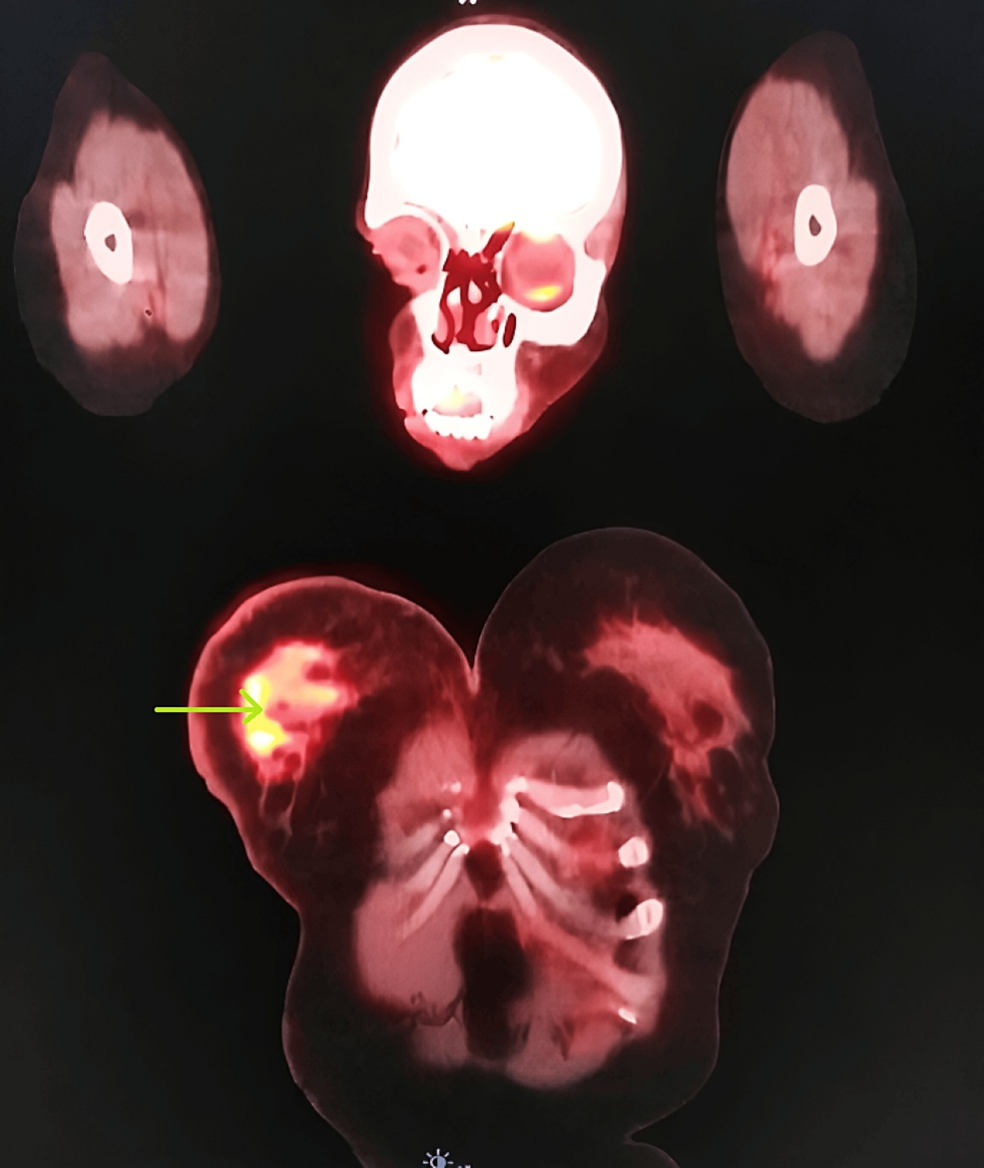
Metastasized breast cancer on PET scan - image 1 Green arrow: metastatic carcinoma in the left axilla PET: positron emission tomography

**Figure 3:**
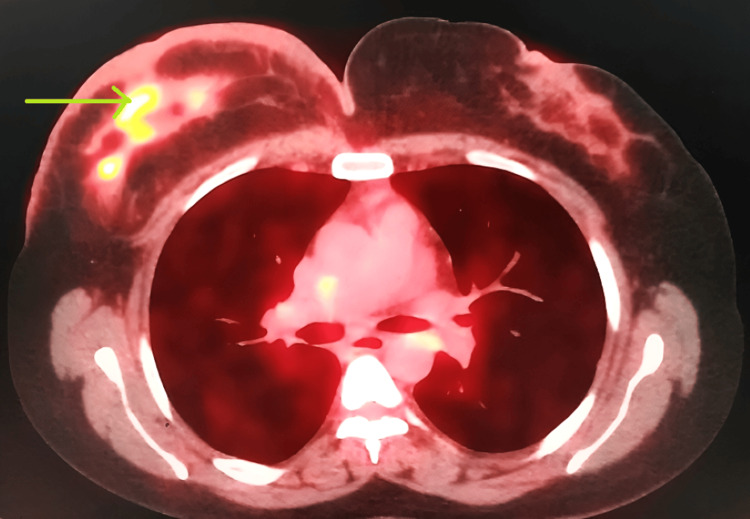
Metastasized breast cancer on PET scan - image 2 Green arrow: metastatic carcinoma in the left axilla PET: positron emission tomography

In early 2019, the patient experienced a cancer recurrence, manifested by swelling in the left axilla. Prompt consultation and ultrasonography revealed lymph node swelling, confirmed through biopsy as metastatic carcinoma. Hormone receptor testing on the recurrent lesion remained positive for ER and PR. A positron emission tomography (PET) scan (Figures 2-3) identified multiple metastases extending to the liver. The patient underwent a full treatment approach, including radiation therapy. After the completion of this treatment, further monitoring and management were implemented.

Laboratory examination

The patient was advised to undergo hysteroscopy, which revealed a normal uterine cavity, healthy cervix and endocervix, and normal bilateral ostia. Afterward, the patient underwent a hysterosalpingography (HSG) examination, confirming a healthy uterine cavity, normal bilateral ovaries, and normal spillage in both fallopian tubes. The patient was then advised to undergo a hormonal profile and CBC. The reports showed a hemoglobin (Hb) level of 10.09 mg/dL. Follicle-stimulating hormone (FSH) was present at a concentration of 9.85 mIU/mL while luteinizing hormone (LH) was detected at a concentration of 12.40 mIU/mL. The anti-Mullerian hormone level was 1.02 ng/mL, and the thyroid-stimulating hormone (TSH) level was 5.42 IU/mL. Prolactin was measured at 40.77 ng/mL (Table 1).

**Table 1:** Hormonal profile of the female patient LH: luteinizing hormone; TSH: thyroid-stimulating hormone; FSH: follicle-stimulating hormone

Parameters	Lab value	Reference range
LH	12.40 mIU/mL	2-15 mIU/mL
TSH	5.42 IU/mL	0.5-5.0 mIU/mL
FSH	9.85 mIU/mL	3.5-12.5 mIU/mL
Anti-Mullerian hormone	1.20 ng/mL	1.0-4.0 ng/mL
Prolactin	40.77 ng/mL	2-29 ng/mL

The sperm count was 40 million per milliliter (M/ml), surpassing the WHO reference value of more than 15 (M/ml). Total sperm motility was measured to be 65%, exceeding the WHO threshold of more than 40%. However, the percentage of nonmotile sperm was 35%, falling below the WHO-recommended level of less than 60%. The pH level of semen was noted at 7.6, within the WHO reference range of 7.2-8.0. The volume of ejaculation was reported to be 1.9 milliliters (ml), which met the WHO criterion of more than 1.5 ml. Morphologically abnormal sperm constituted 85% of the sample, below the WHO cutoff of less than 96%, while normal morphological sperm accounted for 15%, exceeding the WHO minimum of greater than 4%. Table 2 shows the semen analysis of the husband.

**Table 2:** Semen analysis report of the husband WHO: World Health Organization

Semen parameters	Findings	WHO reference values
Sperm count	40 M/ml	>15 M/ml
Total sperm motility	65%	>40%
Non-motile sperm	35%	<60%
pH	7.6	7.2-8.0
Volume	1.9 ml	>1.5 ml
Morphologically abnormal sperm	85%	<96%
Normal morphological sperm	15%	>4%

Timeline and therapeutic intervention

The male partner underwent a course of antibiotics to address leucocytospermia. Specifically, he received ofloxacin tablets at a dosage of 200 mg twice daily for approximately one week. A follow-up semen analysis revealed a significant reduction in the number of pus cells. To enhance sperm quality and minimize sperm DNA fragmentation, the male partner received a combination of medications, including coenzyme Q-10, L-carnitine, L-tartrate, zinc, and multivitamins.

The female patient commenced her IVF treatment in August 2021. Controlled ovarian stimulation (COS) was employed to retrieve multiple eggs. Given the patient's positive estrogen receptors and her history as a breast cancer patient, estrogen-containing drugs were excluded from the ovarian stimulation protocol. Instead, an anti-estrogen agent, letrozole, was administered for approximately 12 days. On August 20, 2021, the first ovum pick-up was conducted, yielding 5 MII and 4 MI oocytes. A fresh ejaculated semen sample from the husband (sperm count: 10 million/ml) was collected, and ICSI was performed, selecting morphologically normal sperm.

Later on, post-fertilization, the dividing embryo cells were meticulously examined over five days. This process resulted in the formation of five blastocysts, of which four were of good quality (2- 4AB, 2- 4AA), and one was of poor quality. On August 25, 2021 (day five), all four blastocysts of good quality were vitrified using liquid nitrogen and vitrification media. Later on the day of the scheduled ET, two embryos (4AA) were thawed using a thawing kit. These embryos (blastocysts) were maintained in hyaluronan-rich media until 10 minutes before transfer. Ultimately, those day-five embryos were transferred into the uterus. A successful beta-human chorionic gonadotropin (beta-hCG) test was conducted on day 14, confirming the positive outcome of the embryo transfer.

Follow-up

The post-embryo transfer follow-up was conducted as required, precisely after 14 days. The thickness of the endometrium was consistently monitored during each visit, ensuring optimal conditions for proper and, predominantly, successful embryo implantation. A beta-hCG test, administered two weeks following the embryo transfer (beta-hCG: 812) served as an evaluative measure to determine the success of the implantation process.

## Discussion

Over the past two to three decades, considerable research has been dedicated to advancing our understanding of the molecular mechanisms and genetic risk factors that influence susceptibility to and development of breast cancer [14]. The introduction of new treatment modalities has notably enhanced cancer survival rates in the last two decades, shifting the focus to quality-of-life considerations for women who have successfully overcome the disease [15]. The utilization of GnRH agonists (GnRHa) as an alternative to hCG in antagonist protocol cycles has gained acceptance in mitigating the risk of ovarian hyperstimulation syndrome. Importantly, this approach does not adversely affect the number or maturation status of collected oocytes. In the context of breast cancer patients, GnRHa is particularly beneficial as it facilitates a rapid reduction in estradiol levels post-oocyte retrieval [16].

Aromatase inhibitors have demonstrated efficacy in reducing estrogen serum levels among postmenopausal women with breast cancer, thereby contributing to decreased mortality and relapse rates. The use of letrozole has been deemed safe in patients undergoing COS, proving effective in reducing estrogen concentration without compromising oocyte yield [17]. However, it is essential to acknowledge that success rates with this approach are comparatively lower than those achieved through cryopreservation of in vivo matured oocytes or embryos, rendering it an experimental procedure [18].

While DNA fragmentation is often overlooked in the evaluation of male infertility, it serves as a critical parameter indicative of infertility and can influence the outcome of assisted reproduction treatments [19]. Elevated seminal bacterial load and increased leukocytes contribute to impaired male fertility parameters, including sperm motility, DNA integrity, acrosome reaction, and molecular structure damage. Both broad-spectrum antibiotics and antioxidant therapy have exhibited promising results in managing infertility in men with leukocytospermia [20].

The supplementation of antioxidants, particularly vitamins C and E, has demonstrated overall benefits. However, caution is advised due to the potential risks associated with over-supplementation and the need for careful consideration of nutrient deficiencies or necessities. This caution stems from the delicate balance required for fertility, encompassing both reduction and oxidation processes crucial for successful fertilization and subsequent pregnancy [21].

## Conclusions

Young females who get diagnosed with breast cancer in the early days of their marriage face challenges in conception as chemotherapy and other treatments for cancer lead to a decrease in ovarian reserve. With the use of aromatase inhibitors like letrozole, estrogen surges can be avoided, and hence individualized approaches may result in a positive outcome for cancer survivors as well. The strategic use of antibiotics and antioxidant therapy further contributed to improving sperm quality in our case, highlighting the importance of addressing male infertility parameters in the overall fertility treatment plan.
